# Exploring the social cognition network in young adults with autism spectrum disorder using graph analysis

**DOI:** 10.1002/brb3.1524

**Published:** 2020-01-23

**Authors:** Roberto Vagnetti, Maria Chiara Pino, Francesco Masedu, Sara Peretti, Ilenia Le Donne, Rodolfo Rossi, Marco Valenti, Monica Mazza

**Affiliations:** ^1^ Department of Applied Clinical Sciences and Biotechnology University of L'Aquila L'Aquila Italy; ^2^ Regional Centre for Autism Abruzzo Region Health System L'Aquila Italy; ^3^ Department of Mental Health of Modena AUSL Modena Modena Italy

**Keywords:** autism spectrum disorder, graph theory, network analysis, social cognition, social functioning

## Abstract

**Background:**

Autism spectrum disorder (ASD) is characterized by an impairment in social cognition (SC). SC is a cognitive construct that refers to the capacity to process information about social situations. It is a complex network that includes distinct components. Exploring how SC components work together leads to a better understanding of how their interactions promote adequate social functioning. Our main goal was to use a novel statistical method, graph theory, to analyze SC relationships in ASD and Typically Developing (TD) individuals.

**Methods:**

We applied graph theory to SC measures to verify how the SC components interact and to establish which of them are important within the interacting SC network for TD and ASD groups.

**Results:**

The results showed that, in the TD group, the SC nodes are connected; their network showed increased betweenness among nodes, especially for the Theory of Mind. By contrast, in the SC network in the ASD group the nodes are highly disconnected, and the efficient connection among the components is absent.

**Conclusion:**

ASD adults do not show SC competencies and functional communication among these skills. Under this regard, specific components are crucial, suggesting they could represent critical domains for ASD SC.

## Graphical abstract

Autism spectrum disorder (ASD) is characterized by an impairment in social cognition (SC). We applied the graph theory to SC measures to verify how the SC components interact and to establish which they are important within interacting SC networks, for Typically Developing (TD) and ASD groups. Results showed that in the TD group, the SC nodes are connected instead, the SC network in ASD group showed that nodes are highly disconnected.

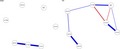

## 1 INTRODUCTION

Autism Spectrum Disorder (ASD) is a heterogeneous neurodevelopmental disorder distinguished by a varied severity of symptoms among affected individuals (Lenroot & Yeung, 2013; Peretti et al., 2019). ASD manifests itself in early childhood and is characterized by a range of deficits in two domains, social communication, and social interaction, in addition to repetitive patterns of behavior (American Psychiatric Association, 2013). According to leading research in this field (Baron‐Cohen, Leslie, & Frith, 1985; Happé, Cook, & Bird, 2017; Mazza et al., 2017; Pino et al., 2017), the central core of autism is the impairment of social cognition (SC) abilities. SC is a complex cognitive process that refers to the capacity of people to store, process and apply information about other people and social contexts (Happé et al., 2017). This ability is essential in our lives, as it allows us to predict other people's behavior and to modify our own behavior in response (Pino et al., 2017; Vetter, Leipold, Kliegel, Phillips, & Altgassen, 2013).

A fundamental component of SC is the Theory of Mind (ToM): The ability to attribute mental (cognitive ToM) and emotional (affective ToM) states to oneself and others, and to use these attributions to make sense of and predict behavior (Baron‐Cohen et al., 1985; Mazza et al., 2017; Warrier & Baron‐Cohen, 2018). We know that during the early months of their lives, individuals with ASD already display impairment of the SC precursors (e.g., emotion processing, sensitivity to ostensive signals, and joint attention, among others (Happé & Frith, 2014; Warrier & Baron‐Cohen, 2018)). According to recent literature (Mazza et al., 2017; Pino et al., 2018, 2017), individuals with ASD are characterized by a delay in the development of SC capacities rather than a total lack of these complex domains/constructs. Indeed, in Typically Developing (TD) children, SC capacities emerge in a specific sequence (Happé & Frith, 2014; Pino et al., 2018, 2017). Individuals with ASD are characterized by the same sequence in the development of these competencies; however, the competencies develop later than in TD children (Pino et al., 2018, 2017).

According to Happé and Frith (2014), SC can be understood as a complex network diagram that includes distinct components such as emotion processing, biological motion perception, empathy, ToM, self‐processing, affiliation, and social identity. All these components are interrelated, and their typical development promotes appropriate social behavior (Happé et al., 2017; Happé & Frith, 2014). Nevertheless their work does not explain how components could interact and how components influence each other. Our study is based on the theoretical framework of Happé and Frith (2014) setting up a simplified model of their network, relying on collected data, which represents the relations between components as well as categorizes graphically their intuition of SC. Thus, SC cannot be considered as a single and independent process, but rather is a complex construct in which the different components work together in an as yet unknown way. Along these lines, a study of the SC components working together could lead to a better understanding of the influences they have on each other, an understanding that would not appear if the components were analyzed in isolation.

A method for evaluating interactions of this kind is graph theory. Indeed, graph theory allows the exploration of associations among interacting elements in a complex network such as the SC domain, the rationale for applying graph theory is that it allows to devise and test structures in terms of components’ connections with the associated path properties (Ibrahim et al., 2016). This type of method can be understood as an ecological approach, in which the SC components are connected together to resolve a social situation and to construct an adaptive response; in fact, the interrelation among the SC components is crucial to developing social behavior and maintaining stable social relationships (Happé & Frith, 2014; Mazza et al., 2017; Pino et al., 2018). In this regard, we suggest that adults with autism, as a result of the delay in the development of SC components (Pino et al., 2018, 2017), could have a dysfunctional or poor social network in which the SC components fail to work effectively to ensure good social functioning.

In this study, therefore, we applied graph theory to behavioral data to verify how the SC components interact and to establish which SC competencies are important within interacting social networks; the theory was applied to both ASD and TD adults, after which the analysis took place. Four SC measures (Basic Empathy Scale; Eyes Task; Empathy Quotient; Advanced ToM Task) were used in this study to evaluate several aspects of the SC constructs ToM, social behavior, and empathy. Specifically, we used these tests because they provide a complete evaluation of mentalizing and empathic abilities. As regards mentalizing tests, the Eyes Task (Baron‐Cohen, Wheelwright, Hill, Raste, & Plumb, 2001) is considered to test the first level of ToM, since it involves the first stage of ToM attribution of the relevant mental state (e.g., compassion) through the observation of the ocular area of face (a visual stimulus). The second stage of ToM attribution involves understanding the content of that mental state (e.g., compassion for a woman who has lost her mother); this is measured by the Advanced ToM Task (Happé, 1994), which evaluates the second stage of ToM through a verbal stimulus. Regarding empathic abilities, the Basic Empathy Scale (Albiero, Matricardi, Speltri, & Toso, 2009; Jolliffe & Farrington, 2006) measures five basic emotions (fear, sadness, anger, and happiness), and the measurements relate more generally to cognitive and affective empathy rather than a specific affective state (e.g., anxiety). The scale is based on the definition of empathy proposed by Cohen and Strayer (1996), as the sharing and understanding of another's emotional state or context resulting from experiencing the emotive state (affective) and understanding the other's (cognitive) emotions. The Empathy Quotient (Baron‐Cohen & Wheelwright, 2004) is more complex; in fact, the authors based this scale on a model in which empathy has both affective and cognitive components. However, some evidence suggests that the scale may consist of three factors (Lawrence, Shaw, Baker, Baron‐Cohen, & David, 2004; Muncer & Ling, 2006) and thus that it also evaluates social skills ability. The Empathy Quotient, compared to the Basic Empathy Scale, evaluates the capacity to share mental states rather than just emotional states.

Using graph theory, we represented the SC components using nodes and their relationships using edges; we then evaluated how these nodes exchanged information and how this differed between ASD and TD groups. We highlight the fact that the qualitative and topographical network differences between atypical and typical development populations could lead to a better understanding of the relationship between social impairments and symptomatology.

## 2 MATERIALS AND METHOD

### 2.1 Participants

Our study included 65 male ASD participants who were selected by the Regional Centre for Autism, Abruzzo Region Health System, L’Aquila, Italy (mean ± standard deviation chronological age = 21.43 ± 2.06), and 61 male TD participants recruited from the University of L’Aquila, Italy (mean ± standard deviation chronological age = 21.52 ± 1.97). No differences between the groups (ASD and TD) emerged for chronological age (*F*_1,124_ = 0.06, *p* = .80).

The ASD diagnoses were provided by experienced clinicians according to the new criteria of the DSM‐5 (American Psychiatric Association, 2013). These diagnoses were confirmed using the Autism Diagnostic Observation Schedule, second edition (Lord et al., 2012) and the Autism Diagnostic Interview—Revised (Rutter, Le Couteur, & Lord, 2003). Given their chronological age, the individuals with ASD and TD were tested with the Wechsler Adult Intelligence Scale (WAIS‐IV; Wechsler, 2008; see Table 1).

**Table 1:** Demographic data for ASD and TD groups and clinical information concerning the ASD group

	TD group (*n* = 61) Mean (*SD*)	ASD group (*n* = 65) Mean (*SD*)	*F*(1,124)	*p*
Demographic data				
Chronological age	21.52 (1.97)	21.43 (2.06)	0.06	.80
Clinical information				
ADOS‐social communication and social interaction	–	8.18 (2.58)		
ADOS‐repetitive and stereotyped behaviors	–	1.47 (1.0)		
ADOS total scores	–	9.71 (3.35)		
ADI‐R reciprocal social interaction		17.60 (4.45)		
ADI‐R communication verbal		10.30 (3.59)		
ADI‐R communication nonverbal		6.30 (3.56)		
ADI‐R restricted, repetitive, stereotyped behavior		5.50 (2.22)		
VIQ	98.00 (23.27)	103.40 (19.72)	1.98	.16
PIQ	95.00 (13.50)	95.60 (12.34)	0.06	.79
TIQ	96.40 (15.23)	97.40 (13.03)	0.15	.69

It was crucial that participants in the TD group had not been diagnosed with any neurological or psychological disorders.

All the participants were tested individually in a quiet room following the principles established by the Declaration of Helsinki. The study was approved by the Ethical Committee of the NHS Local Health Unit (Azienda Sanitaria Locale 1), which approved the experimental protocol prior to the recruitment of participants, according to the principles established by the Declaration of Helsinki. Informed consent was obtained from all the participants before the study.

The socio‐demographic and clinical information for the two groups of participants is summarized in Table 1.

### 2.2 Social cognition measures

#### 2.2.1 Basic empathy scale (*BES)*

The BES is composed of two subscales: the Affective Empathy Subscale (AES) and the Cognitive Empathy Subscale (CES; Albiero et al., 2009; Jolliffe & Farrington, 2006). The AES is composed of 11 items that measure an individual's ability to share another person's emotions. An example of the type of item in the AES is: “My friend's emotions don't affect me much.” The CES comprises nine items and measures the person's understanding of another person's emotions (Jolliffe & Farrington, 2006). Examples of items in the CES are: “I can understand my friend's happiness when she/he performs well in something,” and “When someone is feeling down, I can usually understand how they feel.” The participants had to give their ratings on a five‐point Likert‐type scale ranging from 1 = strongly disagree to 5 = strongly agree. The scores for each item were summed, giving a total score for each subscale (AES and CES) which we used in our analysis.

#### 2.2.2 Eyes task (ET)

The Eyes Task is a revised version of the “Reading the Mind in the Eyes Test”; this test was considered by Baron‐Cohen et al. (2001) to be a first level ToM test. The respondents are given 36 photographs depicting the ocular area of an equal number of different actors and actresses. In the corner of every photograph, four emotional descriptors (e.g., dispirited, bored, playful, or comforting) are printed, only one of which (the target word) correctly identifies the depicted person's mental state, while the others are included as foils. The overall score totals the number of items (photographs) for which the participant correctly identifies the emotional descriptor. The maximum total score is therefore 36. The total score for the Eyes Task was used in our analysis.

#### 2.2.3 Empathy quotient (EQ)

The EQ is a self‐reported measure evaluating different aspects of empathy, using cognitive, social skills, and emotional subscales (Baron‐Cohen & Wheelwright, 2004). The cognitive dimension of empathy is evaluated by three subscales of the EQ: cognitive empathy (CEQ) and social skills (SSQ), which measure, respectively, the capacity to understand the perspective of the other person, and a number of regulatory mechanisms that keep track of the origins of one's own and others’ feelings. The emotional dimension is evaluated by the emotional subscale (EEQ). An example of the items is “I find it hard to understand how to behave in a social situation.” Each answer can vary from 0 (strongly agree) to 4 (strongly disagree). An algorithm permits the responses to be coded according to the response and the item to which it refers, each response in 0, 1, or 2 scores. The item scores are then summed according to their subscales (CEQ, SSQ, and EEQ). The total scores for each subscale were used in our analysis.

#### 2.2.4 Advanced theory of mind task (A‐ToM)

The A‐ToM is an Italian adaptation of a cognitive task that Blair and Cipolotti (2000) used and that was first proposed by Happé (1994). The Italian task consists of an abridged version of 13 vignettes, each accompanied by two questions: the comprehension question “Was it true, what X said?”, and the justification question “Why did X say that?”. The 13 story‐types are Lie, White Lie, Joke, Pretence, Misunderstanding, Double Bluff, Contrary Emotions, Figure of Speech, Appearance/Reality, Forgetting, Irony, and Persuasion. The subject obtains a score ranging from 0 to 1 for each question. A total score, in the range 0–13, is then obtained by summing the scores obtained for each item. We used this total score in our analysis. Happé (1994) used the term “advanced” to refer to a story that contains the comprehension question, where the key questions in the task concern a character's mental state (the experimental condition).

### 2.3 Data analyses

#### 2.3.1 Standard analysis

We performed ANOVA for between‐group comparisons, and the results were adjusted using Bonferroni's correction. The analyses were performed using R (R Development Core Team, 2008).

#### 2.3.2 Network analysis

Graphs give a better way of dealing with abstract concepts like relationships and interactions, and they also provide an intuitive visual way of thinking about these concepts (Kellermann, Bonilha, Lin, & Hermann, 2015; Shirinivas, Vetrivel, & Elango, 2010). According to Ibrahim et al. (2016), the concepts of graph theory make a good method for the analysis of complex networks. In the present study, we used the graph analysis to define the relationships between social cognition domains. In graph theory, the variables are termed “nodes” and they are connected via “edges.” Edges can be weighted, and an edge with a higher weight is more strongly connected with a node than an edge with a lower weight. Moreover, edges can be directed, meaning that the edge between nodes A and B is different from the edge between nodes B and A (Opsahl, Agneessens, & Skvoretz, 2010). In our study, the SC measures constitute the nodes of the network, with the partial correlations between them as weighted and undirected edges. In graph analysis, there are several properties that can be inferred from a network. Some of the canonical centrality indices are represented by *strength*, *betweenness,* and *closeness* (Rubinov & Sporns, 2010).

#### 2.3.3 Network analysis: construction of the network

Two graphs were constructed, one for the ASD group and one for the TD group, with the nodes representing psychological domains (in our study, the nodes represented SC components/abilities) obtained from the assessed tests. We transformed all the scores for the SC measures into *z*‐scores to allow a better comparison of the data. Networks were then estimated using the Gaussian graphical model (Lauritzen, 1996), in which edges can be directly interpreted as partial correlation coefficients using the covariance matrix as the input. This task was carried out using the command *estimate network* (Epskamp, Borsboom, & Fried, 2018) provided by the *bootnet* package running in the R software. Only significant partial correlations were maintained, in order to maintain only important edges.

After the construction of the graph, we evaluated some canonical centrality indices, such as the *strength*, *betweenness,* and *closeness* of each node (i.e., each SC component) (Epskamp et al., 2018).

Specifically, strength represents a weighted measure of the degree between a node and any other node connected to it. It is given by the formula:

$$k_{i}=\sum_{j\in N}w_{\mathrm{ij}}$$

where *k* represents the strength, and *w* represents the weight between the nodes *i* and *j*. We decided to set *w_ij_* as the correlation coefficient between the nodes *i* and *j*. This form of local connectivity defines how much this construct is able to correlate (communicate) with the adjacent nodes.

The need to model the capacity of a node to link to other nodes has been defined using the concept of betweenness, which represents how many times a node is important in the average path between two other nodes:

$$b_{i}=\sum_{j<k}\frac{p_{\mathrm{jk}}\left(i\right)}{p_{\mathrm{jk}}}$$

where *p_jk_* represents the number of shortest paths between nodes *j* and *k*, and *p_jk_*(*i*) is the number of shortest paths between nodes *j* and *k* that pass through node *i*. A node with higher betweenness has a higher number of shortest paths that pass through it.

Closeness represents the average length of the shortest path between the node and any other node:

$$L_{i}^{-1}=\frac{n-1}{\sum_{j\in Nj\neq i}d_{\mathrm{ij}}}$$

where *d* represents the length of the shortest path between node *j* and *i*, and *n* represents the number of nodes. Closeness can be considered to be a measure of how long it takes for a piece of information from one node to reach other nodes. This definition characterizes the strength of the connectivity with all the network nodes, not just the nearest ones. A lower value of this measure represents a lower distance from the node to others, and thus, closeness indicates how central a node is.

#### 2.3.4 Network analysis: groups comparison

In order to evaluate the differences between the TD and ASD networks, we bootstrapped each network 1,000 times, and for each bootstrap, we obtained the strength, betweenness, and closeness for each node. This computation was performed using the *bootnet* command, selecting the replacement option. Statistically significant differences between the measures were then evaluated using the *z* test and were adjusted by Bonferroni's correction (*α* = 0.05). For the analysis, we used the bootnet package (Epskamp et al., 2018) from the R statistical analysis tool (R Development Core Team, 2008).

### 2.4 Ethics approval

Written informed consent was obtained from participants according to the Declaration of Helsinki, and a local ethics committee approved the study.

## 3 RESULTS

### 3.1 ANOVA

One‐way ANOVA was used to test differences between groups (ASD and TD) regarding all the components of the SC measures (ET, BES, EQ, and A‐ToM Task).

The ANOVA for the Eyes Task showed that the ASD group had lower scores than the TD group (*F*_1,124_ = 24.02, *p* < .01). Similarly, the ASD individuals showed difficulties in both the AES (*F*_1,124_ = 79.02; *p* < .01) and CES (*F*_1,124_ = 228.03; *p* < .01) components of the BES, compared to the TD group. Additionally, the ASD group received lower scores than the TD group for the EEQ (*F*_1,124_ = 140.62; *p* < .01), CEQ (*F*_1,124_ = 24.90, *p* < .01), and SSQ (*F*_1,124_ = 1,242.21; *p* < .01) components of EQ. Finally, the ASD individuals showed impaired performance in the A‐ToM Task (*F*_1,124_ = 135.09; *p* < .01) compared to the TD group. The results of these analyses are reported in Table 2.

**Table 2:** Significant differences between groups (ASD and TD) for social cognition measures and canonical properties of graph analysis (betweenness, closeness, and strength) for each node (i.e., social cognition components)

Social cognition measures	Measures score mean(*SD*)		*F*(1,124)	Betweenness mean(*SD*)		*z*	Closeness mean(*SD*)		*z*	Strength mean(*SD*)		*z*
	ASD	TD		ASD	TD		ASD	TD		ASD	TD	
Cognitive empathy subscale of basic empathy scale	28.40 (5.40)	40.67 (3.44)	228.03*	1.23 (3.11)	0.46 (2.26)	−2.00	0.01 (0.03)	0.01 (0.02)	0.00	1.08 (1.15)	0.61 (0.84)	−3.30*
Affective empathy subscale of basic empathy scale	33.30 (6.76)	42.00 (3.68)	79.02*	2.23 (3.77)	0.81 (2.75)	−3.04	0.01 (0.03)	0.01 (0.02)	0.00	1.40 (1.09)	1.46 (0.91)	0.42
Eyes task	19.90 (5.26)	23.64 (2.89)	24.02*	0.95 (3.16)	0.99 (2.97)	0.09	0.01 (0.03)	0.01 (0.03)	0.00	0.92 (1.20)	1.93 (0.86)	6.84*
Cognitive empathy of empathy quotient	9.86 (3.79)	13.04 (3.33)	24.90*	1.19 (3.39)	0.75 (2.60)	−1.08	0.01 (0.03)	0.01 (0.02)	0.27	0.96 (1.11)	0.91 (0.89)	−0.35
Social skills of empathy quotient	4.00 (0.81)	10.83 (1.32)	1,242.21*	0.82 (2.31)	3.17 (4.77)	4.43*	0.01 (0.03)	0.01 (0.03)	0.00	0.87 (1.22)	1.85 (0.94)	6.36*
Emotional empathy of empathy quotient	9.40 (3.59)	16.66 (3.26)	140.62*	1.80 (3.42)	2.44 (4.35)	1.15	0.01 (0.03)	0.01 (0.03)	0.00	1.31 (1.12)	1.55 (0.99)	1.60
Advanced theory of mind	7.60 (3.37)	12.67 (0.51)	135.09*	1.71 (3.71)	5.94 (5.82)	6.12*	0.01 (0.03)	0.02 (0.03)	2.35	1.10 (1.25)	2.51 (0.79)	9.53*

* Significant differences between ASD and TD groups at *p* < .05 (Bonferroni correction).

### 3.2 Visualization of the networks

**Figure 1:**
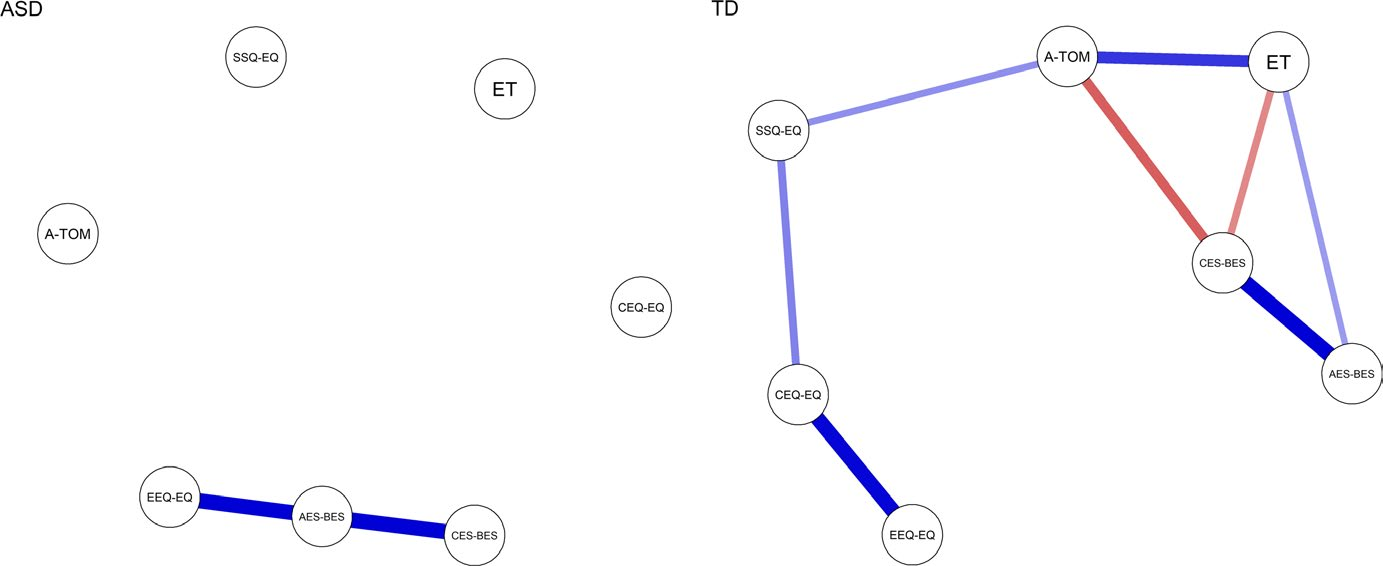
Graphs of ASD and TD populations. Each node represents a SC domain. Strength is represented by the edge's thickness and closeness by the nodes’ distance. Blue and red links represent positive and negative partial correlation coefficients, respectively. AES‐BES, Affective Empathy subscale of BES; A‐ToM, Advanced Theory of Mind task; CEQ‐EQ, Cognitive empathy; CES‐BES, Cognitive Empathy subscale of BES; EEQ‐EQ, Emotional empathy; ET, Eyes Task; SSQ‐EQ, Social Skills

The networks are represented in Figure 1. The nodes represent the different SC components. The lines between the nodes represent the correlations between the measures. The width of the lines indicates how strong a correlation is, while red and blue lines represent positive (blue) and negative (red) correlations. Closeness is represented by the node's distance.

### 3.3 Graph measures

**Figure 2:**
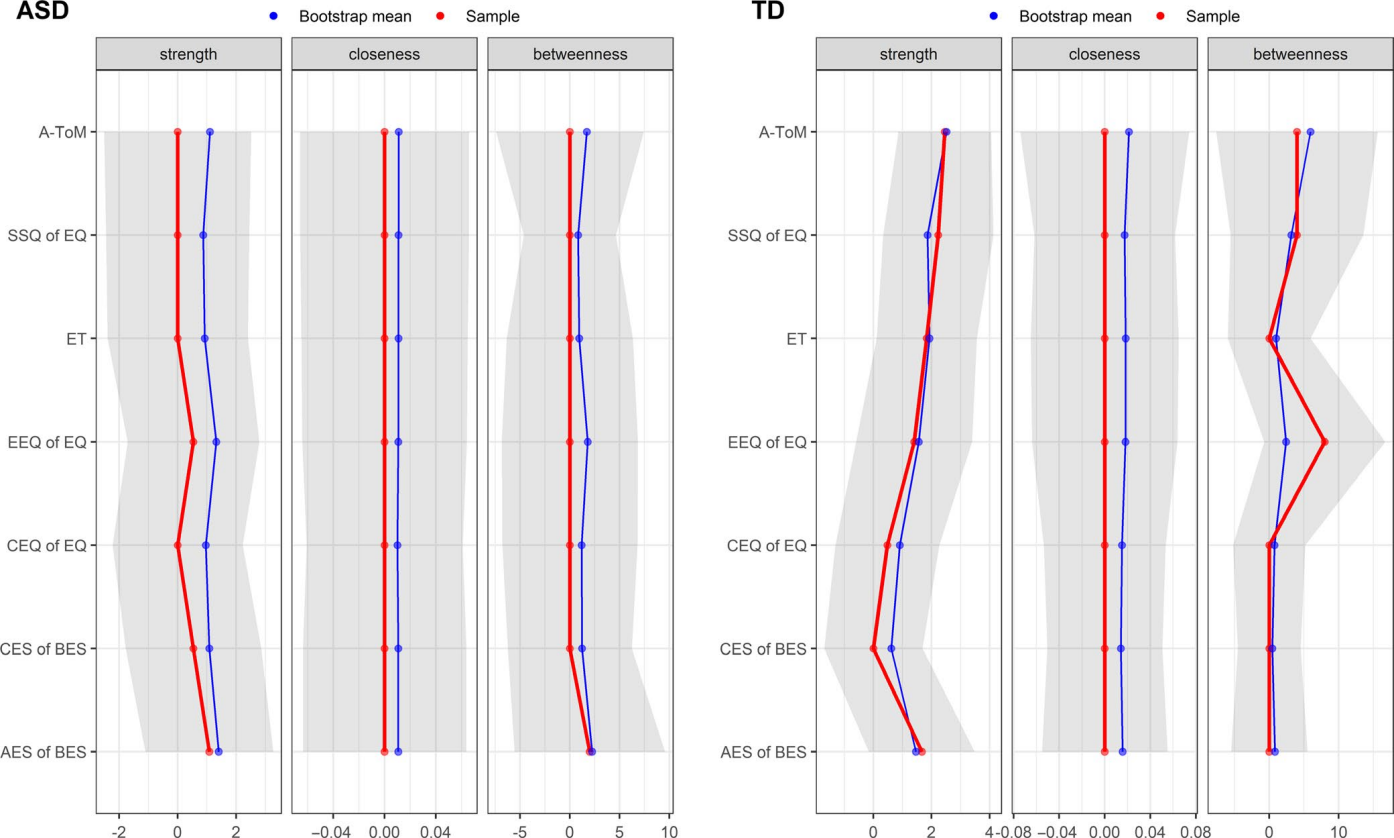
Sample (red line), bootstrap mean (blue line), and *SEM* (gray area) of centrality indices for each node and group

In Figure 2, the centrality indices (strength, betweenness, and closeness) of each node (SC components) for each group (ASD and TD) resulting from the original sample and from the bootstrap are reported. The graph analysis on the centrality indices of each node and between groups showed significant differences between the nodes of the ASD and TD graphs in strength and betweenness properties. No significant differences were found for the closeness property (see Table 2). Specifically, the TD group showed higher betweenness for the SSQ of EQ node (*z* = 4.43, *p* < .01), and higher betweenness for the A‐ToM node (*z* = 6.12, *p* < .01), compared to the ASD group. Moreover, the TD group had higher strength for the ET node (*z* = 6.84, *p* < .01), the SSQ of EQ node (*z* = 6.36, *p* < .01), and the A‐ToM node (*z* = 9.53, *p* < .01), compared to the ASD group.

By contrast, for the CES of BES node, the ASD group showed higher strength (*z* = −3.30, *p* = .02) compared to the TD group.

It is interesting to note that the betweenness for the AES of BES node showed a substantial trend toward being statistically significant, with the ASD group showing higher betweenness (*z* = −3.04, *p* = .05) than the TD group.

The graphs for the ASD and TD groups are reported in Figure 1, and the values of the betweenness, strength, and closeness properties are reported in Table 2.

## 4 DISCUSSION

The aim of this study was to explore the domain of the SC network in young adults with autism, compared with TD adults as a control group, using graph theory. We built two separate networks of SC components, one for ASD and one for TD, in order to map the interacting associations among the components that characterize this construct. Indeed, the SC construct can be defined as a complex network in which interacting components, such as ToM, emotion processing, empathy, self‐processing, and social identity, influence each other (Happé & Frith, 2014; Warrier & Baron‐Cohen, 2018). Individuals with ASD are characterized by a delay in the development of SC competencies (Mazza et al., 2017; Pino et al., 2018, 2017). Consequently, they have difficulty in socializing both with other people and in different social situations (Mazza et al., 2017; Warrier & Baron‐Cohen, 2018); social impairment negatively affects their interpersonal relationships. Happé and Frith (2014) suggest that SC can be understood as a complex network diagram. Thus, based on the theoretical framework of Happé and Frith (2014) we set up a simplified model of their network, which represents the relations between components and categorizes graphically their intuition of SC.

In this regard, we highlight the fact that it is important to evaluate the SC network to determine the relationship among the SC domains and, at the same time, to verify the efficiency of interacting SC networks in young adults with autism to understand how this affects their general social functioning. Therefore, we have outlined, using graph theory, the profile of the SC domain for both typical and atypical populations (TD and ASD groups, respectively).

First, we showed that our individuals with ASD show differences in the SC measures (Eyes Task, BES, EQ, and A‐ToM Task) used in this study, compared to TD individuals. This result is in line with the more recent literature (Mazza et al., 2017; Warrier & Baron‐Cohen, 2018), and it confirms that SC difficulties are a central feature in ASD individuals. For this reason, it has become important to understand how these abilities interact. Graph analysis seemed to be a useful type of statistical analysis for studying the interaction among several of the SC abilities.

Our graph analysis results showed that the SC network is significantly different in the TD and ASD groups. It must be pointed out that our results must be interpreted with some caution, as our analysis describes connectivity between SC domains without specifying how a node processes the information or the direction of the information flow that follows. The first result was that in the TD group all the SC nodes are connected, while in the ASD group the dimensions are highly disconnected (see Figure 1). These results suggest that the SC domains of the ASD network are characterized by poor communication between them, unlike the SC domains of the TD network. Social cognition is a domain characterized by multidimensional components, although if they are effectively involved during social cognition one would expect them to have a relationship that binds them together as a single component. As expected, the high connectivity in the TD network shows that the network works as a single component, social cognition (see Figure 1). An isolated SC network does not mean impaired node processes, but it means that the components do not relate to each other during a social situation. This isolation could be connected to the difficulties expressed by ASD individuals in social situations, where, in order to exhibit appropriate behavior, multiple pieces of information and processes are involved and work in relation to each other. From a behavioral point of view, it is plausible that the isolation of the network in ASD individuals could lead to difficulties in correctly understanding a complex social situation in which more types of information need to influence each other. For example, if ASD individuals are faced with two contrasting sources of information (e.g., a person who smiles even though something bad has happened to him/her), they could treat only one piece of information (e.g., the smile) as being prominent, while the other piece does not contribute properly to their comprehension, failing to lead to the correct interpretation (a fake smile). This could be true even when there are more social clues that complement a correct understanding. Indeed, the SC network of the TD group showed increased betweenness among nodes, specifically the A‐ToM (the complex cognitive capacity) and the SSQ of EQ (social skills abilities) nodes, compared to the ASD group. This finding suggests that these processes represent important hubs of connection that differentiate between ASD and TD individuals when a social situation occurs. Specifically, A‐ToM represents the capacity to understand the perspective of another person, while SSQ of EQ represents a mechanism that keeps track of the origin of one's own and another's feelings. As betweenness represents how paths pass through that node, it is conceivable that these paths support the connection between the cognitive and affective dimensions of empathy (EEQ of EQ and AES of BES) in the network (see Figure 1), allowing information to spread through multiple nodes, although the nature of the support (e.g., monitoring or integrating information) cannot be demonstrated.

Indeed, the TD individuals showed greater strength in the A‐ToM, ET, and SSQ of EQ measures, compared to the ASD group. These results underline the importance of the influence of A‐ToM and SSQ of EQ in the network in combination with ET, suggesting that their information has a major local influence on the TD network compared to the ASD network.

By contrast, the SC network of the ASD group showed that the efficiency of the connection among all the SC components/domains associated with the elaboration of the complex cognitive domain (A‐ToM and SSQ of EQ) is reduced. Indeed, as shown in our results, the ASD network is characterized by increased betweenness, that is, by major functional communication between nodes, in the affective component of empathy (AES of BES), when compared to the TD network. It is plausible that this node could be used as a “compensatory mechanism” during social situations in ASD individuals, although our results only show a trend toward significance (*p* = .05) so we must consider this result with caution. Moreover, the ASD group showed higher strength (i.e., higher activation between local nodes) for CES of BES (cognitive empathy) compared to the TD group, showing a higher locally restricted contribution in the network. Nevertheless, the lack of connections between nodes could be the cause of this restricted contribution in the network. It must be pointed out that in our study ToM measures are performance‐based measures while empathy measures are self‐report measures, so empathy measures evaluate the subject's perception regarding his/her own capacity to share the emotions of other people.

Overall, our results support the idea that young adults with TD have a functional social network, as demonstrated by the fact that all SC competencies communicate with each other and all information is exchanged between different nodes; the existence of a strong betweenness for the cognitive abilities could influence the contribution of the other domains. By contrast, the lack of connection and communication among the SC domains characterizes the ASD network.

Future research should be directed toward understanding how node processes effectively contribute to the network (e.g., by integrating or controlling information) and how edges change over time. It is plausible that these structures are time‐dependent, especially during early development, and it would be interesting to understand when the structure of SC becomes permanent. It would be also interesting to know the level at which the SC network structure can be changed—whether it can be changed topologically or in the magnitude of its edges. Hypothetically, an intervention could try to work on the strength of the edges to improve the network connections, although the poor connectivity in ASD may suggest that intervention in relation to a single component would not be enough to give an important improvement when ASD individuals are facing social situations, because it is possible that any improvements would not influence the SC domain, although future studies should investigate this point.

## 5 CONCLUSION

ASD domains of SC network result isolated compared to TD which results connected, so that the number of connected components changes from one in the TD group into scattered isolated or low connected components in the ASD group. The model obtained from the TD network fits the theoretical SC network model proposed by Happé and Frith (2014), where components are connected to each other. Network indices comparison between TD and ASD group show a different communication role and statistical weight of the SC network nodes. These differences deserve a better understanding and evidence because they could provide strategical indication to impact ASD social functioning as well as their social isolation.

## CONFLICT OF INTEREST

The authors declare that they have no competing interests.

## Data Availability

The datasets generated and/or analyzed during the current study are not publicly available, because the data were obtained in the course of mental health care, but they are available from the corresponding author on reasonable request.

## References

[brb31524-bib-0001] Albiero, P. , Matricardi, G. , Speltri, D. , & Toso, D. (2009). The assessment of empathy in adolescence: A contribution to the Italian validation of the “Basic Empathy Scale”. Journal of Adolescence, 32, 393–408. 10.1016/j.adolescence.2008.01.001 18691746

[brb31524-bib-0002] American Psychiatric Association (2013). Diagnostic and statistical manual of mental disorders (DSM‐5^®^). Washington, DC: American Psychiatric Pub.

[brb31524-bib-0003] Baron‐Cohen, S. , Leslie, A. M. , & Frith, U. (1985). Does the autistic child have a “theory of mind”? Cognition, 21, 37–46. 10.1016/0010-0277(85)90022-8 2934210

[brb31524-bib-0004] Baron‐Cohen, S. , & Wheelwright, S. (2004). The empathy quotient: An investigation of adults with Asperger syndrome or high functioning autism, and normal sex differences. Journal of Autism and Developmental Disorders, 34, 163–175. 10.1023/B:JADD.0000022607.19833.00 15162935

[brb31524-bib-0005] Baron‐Cohen, S. , Wheelwright, S. , Hill, J. , Raste, Y. , & Plumb, I. (2001). The “reading the mind in the eyes” test revised version: A study with normal adults, and adults with Asperger syndrome or high‐functioning autism. Journal of Child Psychology and Psychiatry, 42, 241–251. 10.1111/1469-7610.00715 11280420

[brb31524-bib-0006] Blair, R. J. , & Cipolotti, L. (2000). Impaired social response reversal: A case of acquired sociopathy'. Brain, 123, 1122–1141. 10.1093/brain/123.6.1122 10825352

[brb31524-bib-0007] Cohen, D. , & Strayer, J. (1996). Empathy in conduct-disordered and comparison youth. Developmental psychology, 32, 988 10.1037/0012-1649.32.6.988

[brb31524-bib-0008] Epskamp, S. , Borsboom, D. , & Fried, E. I. (2018). Estimating psychological networks and their accuracy: A tutorial paper. Behavior Research Methods, 50, 195–212. 10.3758/s13428-017-0862-1 28342071 PMC5809547

[brb31524-bib-0009] Happé, F. G. (1994). An advanced test of theory of mind: Understanding of story characters' thoughts and feelings by able autistic, mentally handicapped, and normal children and adults. Journal of Autism and Developmental Disorders, 24, 129–154. 10.1007/BF02172093 8040158

[brb31524-bib-0010] Happé, F. , Cook, J. L. , & Bird, G. (2017). The structure of social cognition: In (ter) dependence of sociocognitive processes. Annual Review of Psychology, 68, 243–267. 10.1146/annurev-psych-010416-044046 27687121

[brb31524-bib-0011] Happé, F. , & Frith, U. (2014). Annual research review: Towards a developmental neuroscience of atypical social cognition. Journal of Child Psychology and Psychiatry, 55, 553–577. 10.1111/jcpp.12162 24963529

[brb31524-bib-0012] Ibrahim, G. M. , Morgan, B. R. , Vogan, V. M. , Leung, R. C. , Anagnostou, E. , & Taylor, M. J. (2016). Mapping the network of neuropsychological impairment in children with autism spectrum disorder: A graph theoretical analysis. Journal of Autism and Developmental Disorders, 46, 3770–3777. 10.1007/s10803-016-2929-8 27696182

[brb31524-bib-0013] Jolliffe, D. , & Farrington, D. P. (2006). Development and validation of the Basic Empathy Scale. Journal of Adolescence, 29, 589–611. 10.1016/j.adolescence.2005.08.010 16198409

[brb31524-bib-0014] Kellermann, T. S. , Bonilha, L. , Lin, K. K. , & Hermann, B. P. (2015). Mapping the landscape of cognitive development in children with epilepsy. Cortex, 66, 1–8. 10.1016/j.cortex.2015.02.001 25776901 PMC4405468

[brb31524-bib-0015] Lauritzen, S. L. (1996). Graphical models. Oxford, UK: Clarendon Press.

[brb31524-bib-0016] Lawrence, E. J. , Shaw, P. , Baker, D. , Baron-Cohen, S. , & David, A. S. (2004). Measuring empathy: reliability and validity of the Empathy Quotient. Psychological medicine, 34, 911–920. 10.1017/s0033291703001624 15500311

[brb31524-bib-0017] Lenroot, R. K. , & Yeung, P. K. (2013). Heterogeneity within autism spectrum disorders: What have we learned from neuroimaging studies? Frontiers in Human Neuroscience, 7, 733 10.3389/fnhum.2013.00733 24198778 PMC3812662

[brb31524-bib-0018] Lord, C. , Rutter, M. , DiLavore, P. C. , Risi, S. , Gotham, K. , & Bishop, S. (2012). Autism diagnostic observation schedule (ADOS‐ 2): manual (2nd edn). Los Angeles, CA: Western Psychological Services.

[brb31524-bib-0019] Mazza, M. , Mariano, M. , Peretti, S. , Masedu, F. , Pino, M. C. , & Valenti, M. (2017). The role of theory of mind on social information processing in children with autism spectrum disorders: A mediation analysis. Journal of Autism and Developmental Disorders, 47, 1369–1379. 10.1007/s10803-017-3069-5 28213839

[brb31524-bib-0020] Muncer, S. J. , & Ling, J. (2006). Psychometric analysis of the empathy quotient (EQ) scale. Personality and Individual differences, 40, 1111–1119. 10.1016/j.paid.2005.09.020

[brb31524-bib-0021] Opsahl, T. , Agneessens, F. , & Skvoretz, J. (2010). Node centrality in weighted networks: Generalizing degree and shortest paths. Social Networks, 32(3), 245–251. 10.1016/jsocnet.2010.03.006

[brb31524-bib-0022] Peretti, S. , Mariano, M. , Mazzocchetti, C. , Mazza, M. , Pino, M. C. , Verrotti Di Pianella, A. , & Valenti, M. (2019). Diet: the keystone of autism spectrum disorder? Nutritional neuroscience, 22, 825–839. 10.1080/1028415X.2018.1464819 29669486

[brb31524-bib-0023] Pino, M. C. , Mariano, M. , Peretti, S. , D’Amico, S. , Masedu, F. , Valenti, M. , & Mazza, M. (2018). When do children with autism develop adequate social behaviour? Cross‐sectional analysis of developmental trajectories. Journal European Journal of Developmental Psychology, 17, 71-87. 10.1080/17405629.2018.1537876

[brb31524-bib-0024] Pino, M. C. , Mazza, M. , Mariano, M. , Peretti, S. , Dimitriou, D. , Masedu, F. , … Franco, F. (2017). Simple mindreading abilities predict complex theory of mind: Developmental delay in Autism Spectrum Disorders. Journal of Autism and Developmental Disorders, 47, 2743–2756. 10.1007/s10803-017-3194-1 28597142

[brb31524-bib-0025] R Development Core Team (2008). R: A language and environment for statistical computing. Vienna, Austria: R Foundation for Statistical Computing.

[brb31524-bib-0026] Rubinov, M. , & Sporns, O. (2010). Complex network measures of brain connectivity: Uses and interpretations. NeuroImage, 52, 1059–1069. 10.1016/j.neuroimage.2009.10.003 19819337

[brb31524-bib-0027] Rutter, M. , Le Couteur, A. , & Lord, C. (2003). ADI‐R. Autism Diagnostic Interview Revised. Manual. Los Angeles, CA: Western Psychological Services.

[brb31524-bib-0028] Shirinivas, S. G. , Vetrivel, S. , & Elango, N. M. (2010). Applications of graph theory in computer science an overview. International Journal of Engineering Science and Technology, 2, 4610–4621.

[brb31524-bib-0029] Vetter, N. C. , Leipold, K. , Kliegel, M. , Phillips, L. H. , & Altgassen, M. (2013). Ongoing development of social cognition in adolescence. Child Neuropsychology, 19, 615–629. 10.1080/09297049.2012.718324 22934659

[brb31524-bib-0030] Warrier, V. , & Baron‐Cohen, S. (2018). Genetic contribution to ‘theory of mind’ in adolescence. Scientific Reports, 8, 3465 10.1038/s41598-018-21737-8 29472613 PMC5823893

[brb31524-bib-0031] Wechsler, D. (2008). Wechsler Adult Intelligence Scale‐fourth edition (WAIS–IV). San Antonio, TX: The Psychological Corporation.

